# MINER: exploratory analysis of gene interaction networks by machine learning from expression data

**DOI:** 10.1186/1471-2164-10-S3-S17

**Published:** 2009-12-03

**Authors:** Sidath Randeni Kadupitige, Kin Chun Leung, Julia Sellmeier, Jane Sivieng, Daniel R Catchpoole, Michael E Bain, Bruno A Gaëta

**Affiliations:** 1 School of Computer Science and Engineering, The University of New South Wales, Sydney, NSW, 2052, Australia; 2 School of Biotechnology and Biomolecular Sciences, The University of New South Wales, Sydney, NSW, 2052, Australia; 3 The Tumour Bank, The Oncology Research Unit, The Children's Hospital at Westmead, Westmead, NSW, 2145, Australia; 4 The Oncology Department, The Children's Hospital at Westmead, Westmead, NSW, 2145, Australia

## Abstract

**Background:**

The reconstruction of gene regulatory networks from high-throughput "omics" data has become a major goal in the modelling of living systems. Numerous approaches have been proposed, most of which attempt only "one-shot" reconstruction of the whole network with no intervention from the user, or offer only simple correlation analysis to infer gene dependencies.

**Results:**

We have developed MINER (Microarray Interactive Network Exploration and Representation), an application that combines multivariate non-linear tree learning of individual gene regulatory dependencies, visualisation of these dependencies as both trees and networks, and representation of known biological relationships based on common Gene Ontology annotations. MINER allows biologists to explore the dependencies influencing the expression of individual genes in a gene expression data set in the form of decision, model or regression trees, using their domain knowledge to guide the exploration and formulate hypotheses. Multiple trees can then be summarised in the form of a gene network diagram. MINER is being adopted by several of our collaborators and has already led to the discovery of a new significant regulatory relationship with subsequent experimental validation.

**Conclusion:**

Unlike most gene regulatory network inference methods, MINER allows the user to start from genes of interest and build the network gene-by-gene, incorporating domain expertise in the process. This approach has been used successfully with RNA microarray data but is applicable to other quantitative data produced by high-throughput technologies such as proteomics and "next generation" DNA sequencing.

## Background

The development of high-throughput technologies for measuring RNA levels and estimating gene expression for large sets of genes has provided a new window into transcriptional regulation. RNA species that vary together under a range of conditions are likely to be under common regulation, and indeed, sets of "co-expressed" genes generated by clustering of microarray expression values have proven useful for identifying potential regulatory elements and transcription factor binding sites [1-5].

This type of analysis has been extended to look for patterns of expression correlation between genes resulting from regulatory relationships, for example increased RNA levels for a transcription factor leading to an increase in the RNA levels of the genes whose transcription is activated by this factor. Several approaches have been proposed to identify potential regulatory relationships, including [6-9]. These regulatory relationships can be visualised as a gene regulatory network graph [10], and this graph, in turn, can be further analysed in terms of global properties [11] and to identify network motifs such as feedforward loops, feedback loops etc [12].

A large number of algorithms based on machine learning and reverse engineering principles have been proposed to infer gene regulatory interactions from microarray data (reviewed in [13-15]). However none of these methods has been very successful, in part due to the large amount of experimental noise in microarray data, which can be particularly problematic for "black box" batch learning methods that infer the most likely gene regulatory network from microarray data with little or no consideration for additional biological information, and keep the human biologist out of the loop. Methods that integrate multiple sources of information (expression levels, biological annotation, protein levels etc) [16-18] are promising but face difficulty in capturing and integrating all the relevant biological information, and their complexity can be prohibitive for the biologist user.

We are proposing an alternative approach based on the philosophy of putting users in control of the process of exploring possible regulatory relationships in an interactive fashion and being able to integrate their biological knowledge with machine learning-based predictions of potential regulatory relationships. The standard paradigm is to visualize the very large networks implicit in high-throughput interaction data, then study sub-network interactions in detail. We invert this, going from individual interactions with target genes to construct a larger network centred on those genes, in an interactive process under biologist control. This approach is used in MINER (**M**icroarray **I**nteractive **N**etwork **E**xploration and **R**epresentation), a web browser-based framework that integrates machine learning of potential regulatory relationships from microarray data, presentation of biological relationships based on Gene Ontology (GO) annotations [19], and integration of multiple analyses into a gene regulatory network model that can be the basis for new hypotheses and experiments. This combination of dependency learning, GO annotation distance and interactive visualisation provides a novel approach for investigating potential regulatory relationships in expression data which can complement standard approaches. MINER has been used by our collaborators to explore different data sets, leading to the identification of potential relationships that were subsequently validated experimentally.

## Results

### Interactive exploration of potential regulatory relationships

**Figure 1:**
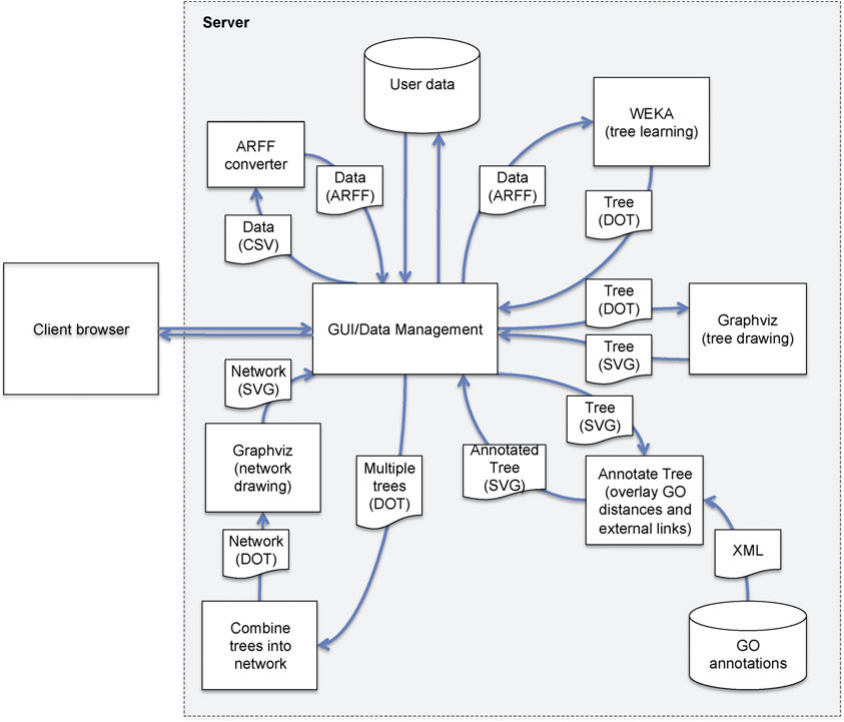
**MINER system-level data flow**. Overview of data exchange between the various components of MINER. All the data passed to the Data management process are stored in the User data database.

**Figure 2:**
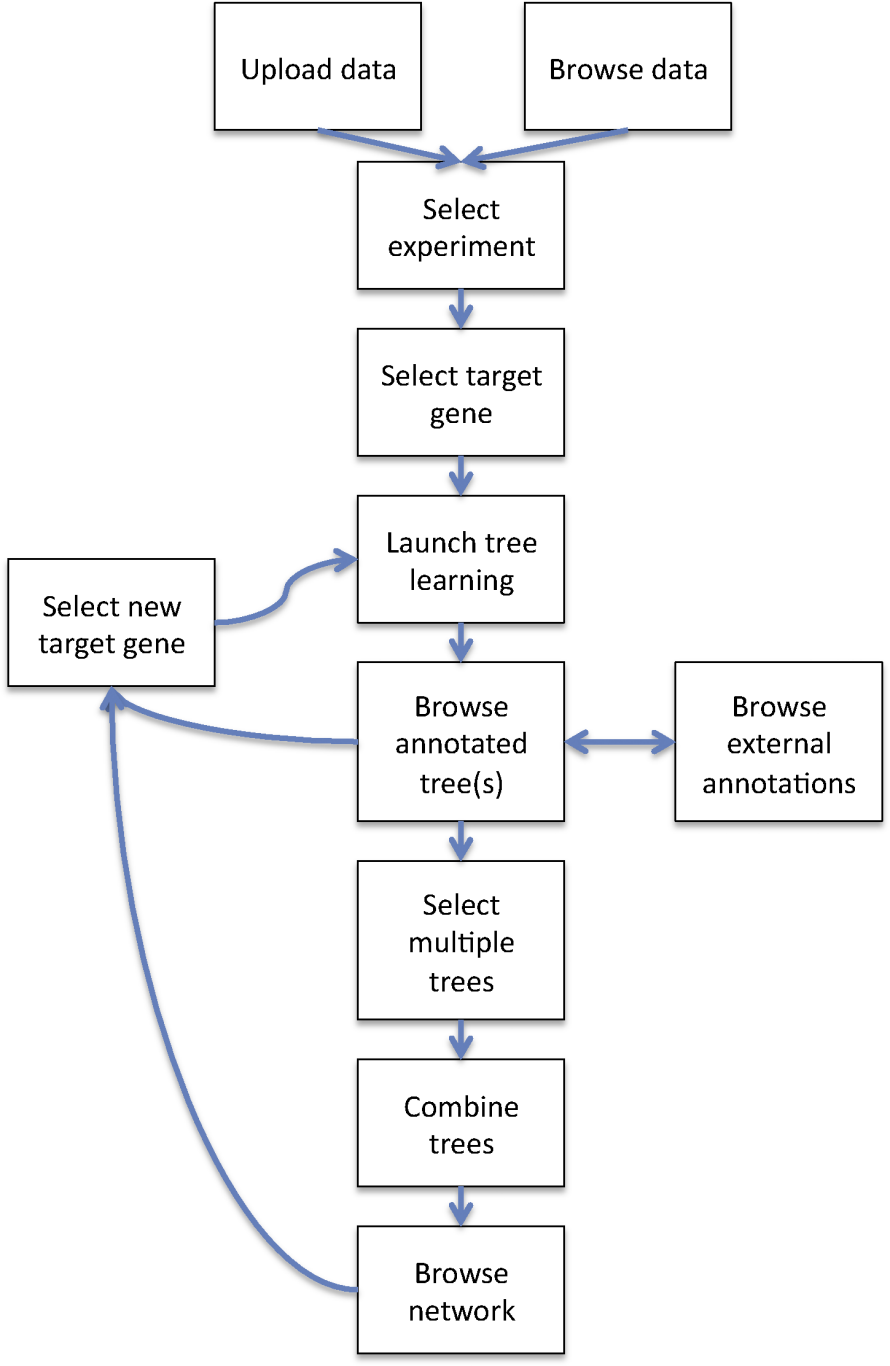
**Typical user workflow in MINER**. Flowchart demonstrating the actions taken by the user in a typical MINER analysis. The user interface guides the user through the various steps. The user introduces biological expertise in the network inference process by choosing genes and trees to further analyse.

**Figure 3:**
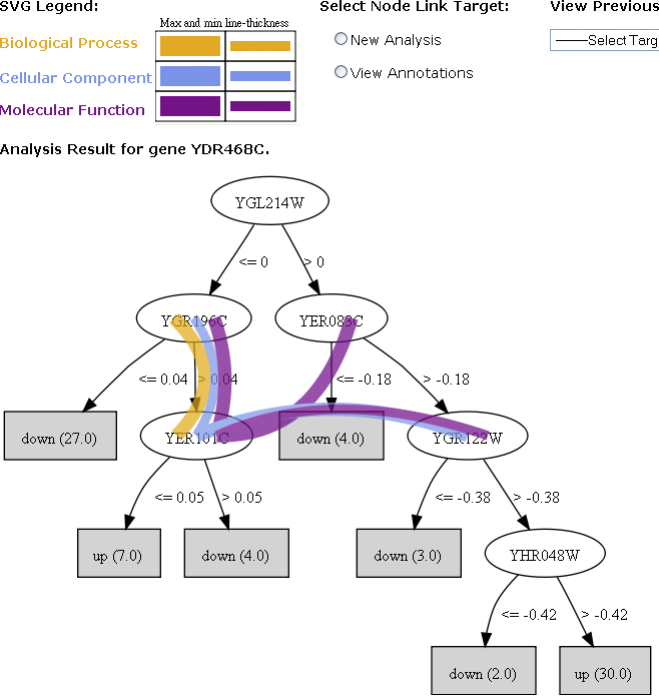
**Example annotated tree (Arctree) produced by MINER**. Screenshot from the MINER GUI showing a decision tree of genes whose expression in the input microarray data set can predict the expression of the target gene YDR468C. For example, in this case, the tree indicates that YDR468C was down-regulated in the data set when the expression of YGL214W had a normalised expression level of 0 or less and YGR196C had a normalised expression level of 0.04 or less. Placing the mouse over a node of the tree displays coloured arcs representing the "annotation distance" between the gene represented by this node and other genes in the tree. Genes who share more GO annotations are linked by thicker arcs. The colour of the arc corresponds to the GO category represented (Biological Process, Molecular Function, Cellular Component). For example, in this case, YGR196C and YER101C share similar GO function, process and localisation annotations. Depending on the radio button setting, clicking on a node either displays external annotations on the corresponding gene or launches a new tree learning analysis using the corresponding gene as the new target.

MINER is a web-based framework that analyses microarray data to suggest likely hypotheses regarding regulatory relationships between genes surveyed in the dataset. The system-level data flow of MINER is shown in Figure 1. The system is fully user-driven and provides a convenient interface and visualisation paradigm that allows the biologist to explore the dependencies and relationships of genes of interest. A typical user workflow is shown in Figure 2. The user uploads normalised microarray data in tabular or colon-delimited format, and then selects one or more target genes of interest in the dataset to launch the analysis. MINER applies decision tree, model tree or regression tree learning [20] to identify genes in the dataset whose expression can predict the expression of the target gene, and displays the result in the form of a decision tree for the user to explore. The user can mouse-over the nodes of the trees to display potential relationships based on common Gene Ontology [19] annotations between the node and other nodes in the tree, in the form of an overlaid graph (figure 3). This visualisation is based on the ArcTree paradigm [21]. "Distances" between genes are calculated based on the graph distance between their GO annotation terms calculated using the Czekanowski-Dice formula [22]. The distance between two genes is represented by the thickness of the line connecting the two corresponding nodes in the tree, and the colour of the line represents the GO category (Molecular Function, Biological Process, Cellular Compartment) represented. Clicking on a node allows the user to display linked annotations in the Kyoto Encyclopaedia of Genes and Genomes (KEGG) database [23] or to launch a new analysis using the selected node as target gene, to build a new tree for this gene.

**Figure 4:**
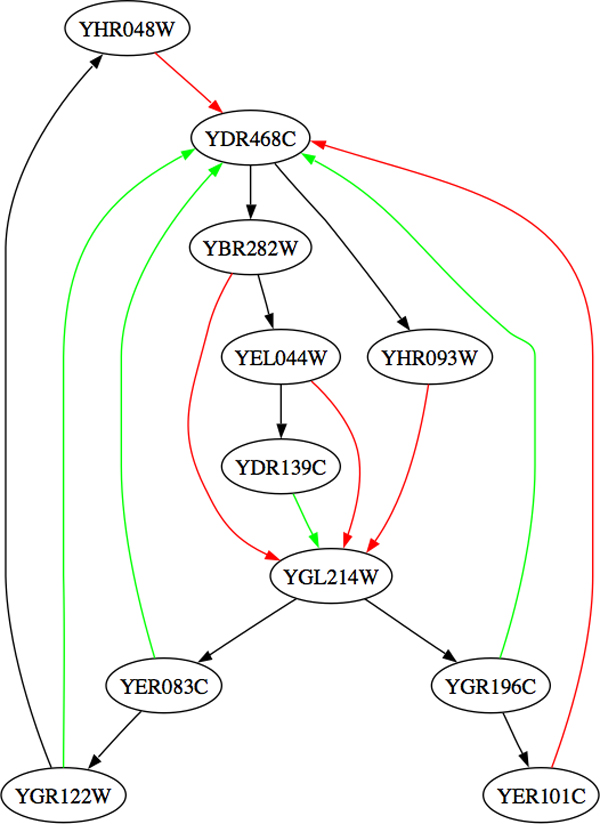
**Example MINER gene interaction network**. Screen capture from MINER showing a section of a gene interaction network generated by MINER from two trees, one of which appears in Figure 3. Regulatory relationships identified in the learned tree are represented as edges in the network, with interactions shown in either red (for up-regulation) or green (for down-regulation) acting on a *target *gene; edges shown in black are between two *non-target *genes and indicate that there *may *be a regulatory relationship or interaction between these genes but the nature of this relationship is not inferable from the component trees.

**Figure 5:**
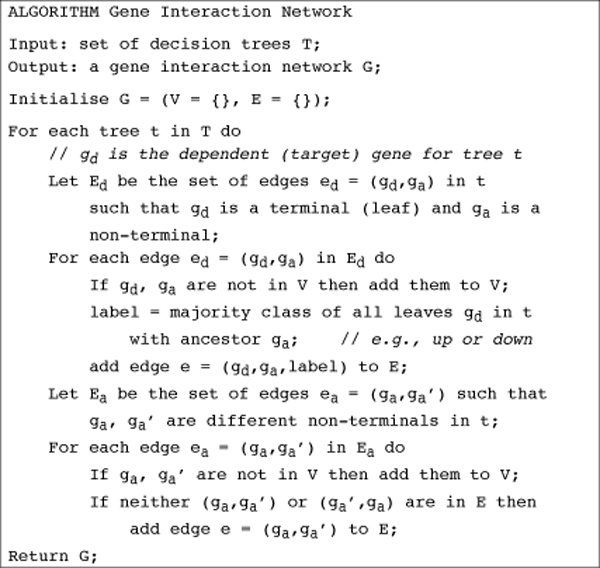
**Algorithm: Gene Interaction Network from Decision Trees**. MINER uses this algorithm to construct a Gene Interaction Network from a set of Decision Trees. Both input trees and the output network are vertex- and edge-labelled directed graphs where vertices represent conditions on genes. However, in each type of graph the vertex and edge label sets differ.

After decision trees have been built for multiple genes, MINER allows summarising the multiple trees into a network graph, by representing each potential regulatory relationship seen in a decision tree as an edge in a graph (figure 4), based on the algorithm given in figure 5. These decision trees, networks and overlaid annotations can assist the user in the formulation of new hypotheses regarding the regulation of the target gene, which can be subsequently tested experimentally.

The tree-learning approach was inspired by the work of Brazma and others [24] and was extended by us to work on real-valued data using regression and model trees [25] where it was applied to yeast microarray data. Further extensions, particularly the use of a relational database, graphical user interface, support for gene interaction network construction and Gene Ontology distance functions were implemented in a number of follow-up projects.

### Evaluation

Due to the large data requirements the MINER system is currently not publicly available on the web but it has been implemented for two of our collaborators, in one case for *S. cerevisiae *data, and in the other for an acute lymphoblastic leukaemia microarray dataset [26]. In the latter case, MINER suggested a new significant regulatory relationship in leukemic cells that was subsequently validated experimentally (Guo D, O' Sullivan M, Henry M, Fong A, Kiiveri H, Stone G, Randeni H, Gaeta B, Bain M and Catchpoole D - manuscript in preparation).

MINER relies on human intervention to guide the network-building process and as such cannot be evaluated in comparison to fully automated "one-shot" network inference algorithms. However, as part of previous work [25] we evaluated the tree learning methods used in MINER on the standard yeast cell cycle microarray data set [27]. In our study three methods of tree learning were used: decision tree learning, where the dependent variable is discrete-values, and two methods of numeric prediction, regression and model trees. All systems were implemented in the WEKA toolkit [20] and learning performance was estimated using 10-fold cross-validation. Tree learning was performed for each of the twenty target genes identified by Soinov et al. [24].

For decision tree learning we found a mean accuracy of 72%, with twelve out of twenty trees scoring above 70% accuracy, and all scoring above 50%. Correlations were above 0.7 for five (resp. eight) out of twenty for regression (resp. model) trees, with mean correlations of 0.5 (resp. 0.6) over all twenty target genes. Given that the data is noisy with a low number of samples, high number of genes, and many missing values, these results are as expected.

Subsequent experiments (unpublished data) using a network simulator to generate synthetic microarray data with artificial noise has shown that the tree learning in MINER can recover the gene dependencies embedded in simple network motifs such as feed-forward loops. In [25] we also found network links between genes across different trees, as would be discovered in automatic construction of gene interaction networks.

## Discussion

### Representing interaction networks

As is common in current genome-scale informatics, the fundamental object for MINER is the "gene", although this can actually refer variously to gene products such as transcripts, proteins, intergenic (promoter) regions, etc. A network may be formalised as a graph *G *= (*V*, *E*), where each vertex in the set *V *denotes a gene, and each edge in the set *E *represents some kind of interaction between genes. Edges may be directed or undirected, and may have labels, e.g., to distinguish between different types of interactions.

### Using a machine learning toolkit

MINER uses the WEKA machine learning toolkit [20] for tree learning. The advantage of a general-purpose machine learning toolkit in the exploratory analysis of genome-scale interaction data is the ease and rapidity with which many different forms of data mining can be performed. For example, it is possible to move quickly from simple visualizations of the data and summary statistics to sophisticated methods such as non-linear multi-variate regression or high-dimensionality kernel-based classifier learning.

Since the predominant mode of analysis in MINER is exploratory rather than hypothesis testing, it is necessary to have powerful methods capable of detecting the faint signals present in noisy data such as microarrays. Although these may increase the risk of Type 1 errors (i.e., false positives, suggesting interactions which in fact have no biological basis), it is understood that any detected interaction will be subject to further analysis by different techniques before they can be accepted. There is also a role in this process for ***integrating ***potential interactions with other sources of data to increase confidence. On the positive side there are many advantages in reverse engineering networks by interactively tracing out patterns of influence of genes on other genes using the powerful means of signal detection implemented in machine learning methods.

Non-linear regression of multiple genes on a target using model tree learning subsumes techniques such as correlation-based construction of co-expression matrices. This is important since regulatory relationships may be non-linear. In particular, this representation can learn context-dependent (potentially regulatory) relationships: as an example, we could have that given gene *A *> 1.3 and gene *B *< -0.9 then the dependence of genes *C*, *D*, and *E *on target *F *is given by the linear regression equation *F *= *-0.2 C + 2.3 D + 0.1 E + 0.7*. Such context sensitivity has the potential to detect regulatory signals in data that could be missed by simply finding the pairwise correlations of genes *A*, .., *E *with target gene *F*.

Tree learning methods also perform attribute (variable) selection during the learning process, finding a subset of genes implicated in potential regulatory relationships with a target, enabling inspection by a biologist, since typically this represents only a small subset of the whole genome. The potential for overfitting can be controlled by user-driven pruning built into the algorithms. Other learning methods such as high-dimensionality kernel methods can be applied to the same data sets; in this case feature selection can be applied by either pre-processing the data, or post-processing the learned model [28].

### Network construction from multiple trees

Transforming a set of trees (e.g., see Figure 3), each of which encodes a disjunction of conjunctive rules on the conditions (gene expression levels) under which a single *target *gene is expressed, to a network that captures the combination of regulatory dependencies between multiple genes in a user-friendly way is not straightforward. We adopted a level-wise approach (Figure 5). At the first level all the trees learned from the expression data are retained, since they capture the details of the regulatory relationships of genes on their targets. A higher-level network is then constructed by combining the trees at the first level and removing some of the detail. Recall that both levels are expanded only as the user explores the space of target genes.

At the network level, the goal is not to provide the detailed logic of combinations of condition-specific gene regulation, but rather to show the general organisation of regulatory gene interactions. To do this we use the structure of the trees. Parents of terminal (leaf) nodes are more closely linked with their target genes and edges are labelled to denote the principal regulatory effect (e.g., up or down). Edges linking non-terminal (internal) nodes are then added without labels to denote an indirect regulatory interaction. Note that functionally these relationships may be just as important. However, this structures the network and reduces clutter in the visualization. Since all details are retained in the trees at the lower level, no information is lost. An example of such a network is shown in Figure 4.

### Integration of heterogeneous data sources

Gene Ontology: MINER uses a distance measure on the GO annotation of pairs of genes [22] to evaluate their biological relatedness. This is currently implemented at the level of individual trees, but could be easily incorporated into network edges as well.

Kyoto Encyclopedia of Genes and Genomes (KEGG): each gene appearing in an internal node of a decision tree is annotated with a species-specific URL denoting its entry in the KEGG GENES database. This is then included in the SVG file that displays the decision tree graphically in the browser interface. When the user clicks on a node in the tree, the browser executes a query to open the gene's annotation page and display details of its name, sequence, and other annotation using KEGG's DBGET method.

Other sources of expression data: Since tree induction methods are non-parametric they may be applied to other data sources, as long as they are in a similar format to mRNA expression data, such as data from next-generation sequencing data, proteomics or glycomics. This is because data generated in the form of (absolute or relative) abundances, such as from high-throughput mass spectrometry are similar to microarray data in the sense of being an indirect measure of concentration of gene products or other molecules. However, this is left for future work since so far we have only applied MINER to microarray data.

### Related work

A large number of methods have been proposed to infer whole gene regulatory networks from gene expression data (reviewed in [13-15]). These methods all apply a "one-shot" paradigm that can lack transparency for the end user and does not allow the use of the biologist's domain knowledge. MINER differs from most approaches through its interactivity that allows the user to explore the data and generate testable hypotheses in the process.

Other interactive methods fall into two categories: network visualisation tools that can incorporate some network inference algorithm, and interactive data mining applications.

In the first category, SEBINI [29] is designed to be a *framework *to support testing of network inference algorithms using synthetic and other data sets. However, it has a limited number of inference methods incorporated, and cannot support the two level approach we have adopted. It also does not seem to be actively under development. ToPNet [30] adopts the Petri Net formalism to represent interactions, which is more flexible than simple graphs, particularly for metabolic reactions. However, it does not support any data mining methods for network inference, and it is no longer supported. Cytoscape [31] is a widely used visualisation and integration package that supports some network inference plug-ins (for example [32,33]). All of these plug-ins perform a global network inference based on uni-variate correlation rather than the gene-by-gene approach of MINER that uses more involved multi-variate non-linear tree learning.

In the second category, SysNet [34] combines visualization and exploratory data analysis, however its network inference is restricted to standard methods of correlation. Unlike MINER, SysNet infers a global network first then allows the user to drill down to inspect properties of individual nodes rather than building the network from individual relationships.

## Conclusion

MINER combines advanced machine learning techniques with a "bottom-up" interactive approach to inferring gene interaction networks from gene expression data. This approach differs from most methods that attempt to reconstruct the whole network in one operation and are not very transparent to the end user, and from interactive methods that are based on relatively simple expression correlation and clustering. The MINER approach allows biologists to examine the program's hypotheses as they are generated and incorporate their own biological knowledge into the interaction network exploration process. The tree learning paradigm provides explicit descriptions of regulatory dependencies with supporting evidence for the user to examine. This interactive exploration approach has already resulted in the discovery of new regulatory relationships that were subsequently validated experimentally. MINER has been used with gene expression data obtained from microarray experiments but can be applied to any high-throughput molecular abundance data including those resulting from new sequencing technologies and from proteomics analyses.

## Methods

### Component packages

MINER is implemented using PHP [35] with some components in Perl [36] It uses the MySQL RDBMS [37] for storing user data, results and GO annotations and relationships. The decision tree learning component of MINER uses the J48 algorithm implemented in WEKA (version 3.4.8) [20] with default parameters (C = 0.25, M = 2). Regression and model tree learning uses WEKA's M5Prime implementation with default parameter settings. Tree and network diagrams are produced using the Graphviz package [38]

### Data formats

Microarray data can be uploaded to MINER in tabular or comma-delimited format, and are converted into ARFF (Attribute-Relation File Format) for input into WEKA. Trees are produced by WEKA in DOT format and converted by Graphviz into images in SVG (Scalable Vector Graphics) format [39] for interactive visualisation. Since MINER's graphical outputs (trees and networks) are in the SVG format, a suitable browser rendering component is required for visualization. Current versions of all major web browsers except Microsoft's Internet Explorer have built-in support for rendering SVG graphics. Users of Internet Explorer can download a plugin to enable SVG support.

### User interface design methodology

The MINER graphical user interface was developed using standard UI development methodology. A range of visualisation paradigms were proposed and non-functional mock-ups were developed. The mockups were presented to a focus group of potential end users whose feedback guided the selection and refinement of the final visualisation paradigm. The design process applied human-computer interaction and ergonomics principles. For example, colours were selected to be easily distinguished even by most colour-blind users.

## List of abbreviations used

GO: Gene Ontology; KEGG: Kyoto Encyclopaedia of Genes and Genomes; ARFF: Attribute-Relation File Format; SVG: Scalable Vector Graphics.

## Competing interests

The authors declare that they have no competing interests.

## Note

Other papers from the meeting have been published as part of *BMC Bioinformatics* Volume 10 Supplement 15, 2009: Eighth International Conference on Bioinformatics (InCoB2009): Bioinformatics, available online at http://www.biomedcentral.com/1471-2105/10?issue=S15.

## Authors' contributions

SRK implemented the basic functionality of the program including decision trees and network visualization. KCL implemented annotated tree visualization, model trees and regression trees. JSe gathered software requirements and designed and tested the visualization paradigm. JSi contributed to the programming and implementation of the system. DRC provided data sets for testing and user feedback. MEB and BAG collaborated on the conception of the project, led the group and drafted the manuscript, with MEB focusing on machine learning analysis and BAG on user interface and implementation. All authors read and approved the final manuscript.
